# Application of the Total Nutrient Index as a Precision Nutrition Tool to Address Dietary Recommendations Across the Life Course

**DOI:** 10.1016/j.jand.2026.156368

**Published:** 2026-04-25

**Authors:** Alexandra E. Cowan-Pyle, Janet A. Tooze, Lindsay M. Reynolds, Jaime J. Gahche, Johanna T. Dwyer, Diane C. Mitchell, Raymond J. Carroll, Bani K. Mallick, Regan L. Bailey

**Affiliations:** A. E. Cowan-Pyle is research faculty III, Institute for Connecting Nutrition and Health and Department of Behavioral Sciences and Social Medicine, College of Medicine, Florida State University, Tallahassee. J. A. Tooze is a professor, Department of Biostatistics and Data Science, Wake Forest School of Medicine, Wake Forest University, Winston-Salem, NC. L. M. Reynolds is an assistant professor, Department of Epidemiology and Prevention, Wake Forest School of Medicine, Wake Forest University, Winston-Salem, NC. J. J. Gahche is a nutritional epidemiologist, Office of Dietary Supplements, National Institutes of Health, Bethesda, MD. J. T. Dwyer is a professor, Jean Mayer USDA Human Nutrition Research Center on Aging, Tufts University, Boston, MA. D. C. Mitchell is a program director, Institute for Advancing Health Through Agriculture, Texas A&M AgriLife Research, Texas A&M University System, College Station. B. K. Mallick is a distinguished professor, Department of Statistics, Texas A&M University, College Station. R. L. Bailey is director, Institute for Connecting Nutrition and Health, and a professor, Department of Behavioral Sciences and Social Medicine, College of Medicine, Florida State University, Tallahassee.

**Keywords:** Total Nutrient Index, Micronutrients, Dietary Supplements, Dietary Reference Intakes, NHANES

## Abstract

**Background:**

Indexes are a standardized tool for assessing dietary exposures from foods/beverages (F&B) and have recently been extended to include dietary supplements (DS). The Total Nutrient Index (TNI) (F&B + DS) and the Food Nutrient Index (FNI) (F&B only) were developed to assess micronutrient intakes relative to Dietary Reference Intakes for micronutrients and were previously examined for relative validity among US adults.

**Objective:**

This study examined the micronutrient quality (ie, TNI and FNI) of the diet across all age groups and compared TNI and FNI total and component scores with Healthy Eating Index 2020 scores among the US population. This analysis also sought to demonstrate the flexibility of the TNI and FNI frameworks across the life course.

**Design:**

A nationally representative, cross-sectional analysis of the 2015-2020 National Health and Nutrition Examination Survey demographic, dietary, and dietary supplement data (ie, 24-hour dietary recall and DS inventory) was conducted among the US population.

**Participants and setting:**

This study included US adults and children (aged 1 year and older; n = 19 903) who participated in the 2015-2020 National Health and Nutrition Examination Survey.

**Main outcomes:**

The main outcome measures were TNI and FNI total and component scores (range = 0 to 100) overall and by age group, with higher scores indicating greater adherence to the Dietary Reference Intakes.

**Statistical analyses performed:**

TNI and FNI scores were calculated via the simple algorithm method, for 11 age group-dependent micronutrients (calcium, choline, magnesium, phosphorus, potassium, and vitamins A, B12, C, D, E, and K), and compared with Healthy Eating Index 2020 scores.

**Results:**

Americans scored 72 out of 100 on TNI, but scores varied by age and were higher for the TNI than FNI (~3 to 11 points.). Younger children (aged 1 to 3 years, TNI = 87; aged 4 to 8 years, TNI = 82) and older adults (aged 71 years and older, TNI = 77) exhibited higher TNI scores than other ages, due to higher calcium, magnesium, and vitamins A, C, and B12 intake. Healthy Eating Index 2020 total scores were low (52) overall and followed similar scoring trajectories by age group as the TNI and FNI.

**Conclusions:**

Evaluating total dietary exposures is important, considering the differential intake patterns from F&B, vs those with DS. Given low Dietary Reference Intakes adherence for some nutrients across the life span, these findings warrant improved diet quality and micronutrient density for many, to optimize nutrition and reduce diet-related chronic disease risk among Americans.

The dietary patterns-based approach is the mainstream analytical method for describing the totality of dietary and nutrient exposures, as well as their effects on human health.^1-6^ This approach is commonly used to describe and quantify dietary exposures and combinations of foods and beverages (F&B) consumed, compare dietary patterns across population subgroups, and to relate these patterns to health outcomes^1-6^ and risk of mortality.^7-9^ Several methodological approaches to dietary pattern analysis exist, yet indexes and scores serve as the hallmark method for characterizing patterns of intake because they are constructed based on predefined criteria and can be compared with a criterion-based standard.^10-12^ The Healthy Eating Index (HEI) is a validated, gold standard measure for evaluating diet quality from F&B and utilizes an energy density-based approach for nutrients or food components (NOFC) to encourage (eg, nutrient-dense foods, like whole fruits and total vegetables) and those to limit (eg, added sugars and sodium).^13^

Although much is known about dietary indexes from F&B,^14-16^ much less is known about patterns of micronutrient exposures and their potential relation with health. As the field of nutrition science has evolved, it has extended beyond the prevention of deficiency disorders and toward reducing the risk of diet-related chronic disease,^17-19^ requiring a measure that not only complements F&B-focused metrics, but also reflects patterns of micronutrient intakes (or other bioactive food components).^20^ Micronutrient density may be particularly relevant to such diet-health associations, given the high prevalence (48%) of micronutrient-containing dietary supplement (DS) use among the US population,^21^ as well as the large contribution of nutrients from this source in some population subgroups.

To better understand the micronutrient density of dietary patterns in the United States, our investigative team recently developed and validated the Total Nutrient Index (TNI), a scoring system inclusive of micronutrients obtained from all sources (ie, F&B and DS) that evaluates nutrient intakes relative to the Dietary Reference Intakes (DRI)^22^ among US adults. The TNI was designed to focus on underconsumed micronutrients in the American diet at the population level^23,24^ and was developed in tandem with the Food Nutrient Index (FNI),^23,24^ an index calculated identically to the TNI, but from dietary sources only (ie, F&B). Recent studies employing the TNI and FNI framework have demonstrated the utility of the TNI for examining the totality of micronutrient exposures,^25-31^ as well as their relation to morbidity and mortality,^32,33^ in various population subgroups, including adolescents, prostate cancer survivors, and community-dwelling older adults.

Nevertheless, because micronutrient requirements vary by age^34,35^ and physiological state,^36^ precision nutrition approaches require identification of nutrients of public health relevance across the life course. Existing diet quality metrics, like the HEI-2020, have also shown that dietary quality from F&B differs depending on age group (HEI-2020 range = 49 to 64 out of 100).^13^ Thus, micronutrient quality may follow a similar age-specific pattern and has the potential to be exacerbated when DS are included in the metric. Hence, a logical extension of the TNI framework was to incorporate a life course-based approach, by characterizing nutrient exposures overall and across all age groups, alongside that of the complementary food- and nutrient-based HEI. In turn, this analysis aimed to examine the micronutrient quality of dietary intakes (ie, F&B only, and F&B + DS) among the US population (aged 1 year and older; n = 19 903) in the National Health and Nutrition Examination Survey (NHANES) 2015-2020, as assessed via the TNI and FNI, among sex- and age-specific groups across the life course and to compare TNI and FNI total and component scores to HEI-2020 scores. This analysis also seeks to demonstrate the adaptability of the TNI framework for different research questions, population subgroups, life course groups, and nutrition policy needs.

## METHODS

### Study Design

NHANES is a nationally representative, cross-sectional, population-based survey conducted by the US Centers for Disease Control and Prevention, National Center for Health Statistics, that monitors the health and nutritional status of the United States.^37^ NHANES employs a complex, stratified, multistage probability cluster sampling design and publicly releases all surveys, available in 2-year datasets.^38,39^ NHANES data collection occurs through an initial in-home interview, an in-depth health evaluation in a mobile examination center, and through a follow-up telephone interview.^37,38^ Written informed consent was obtained from all participants and/or their proxies, and the Research Ethics Review Board at the National Center for Health Statistics approved the NHANES study protocol (as well as the publicly released de-identified data).^40^

**Figure 1.**
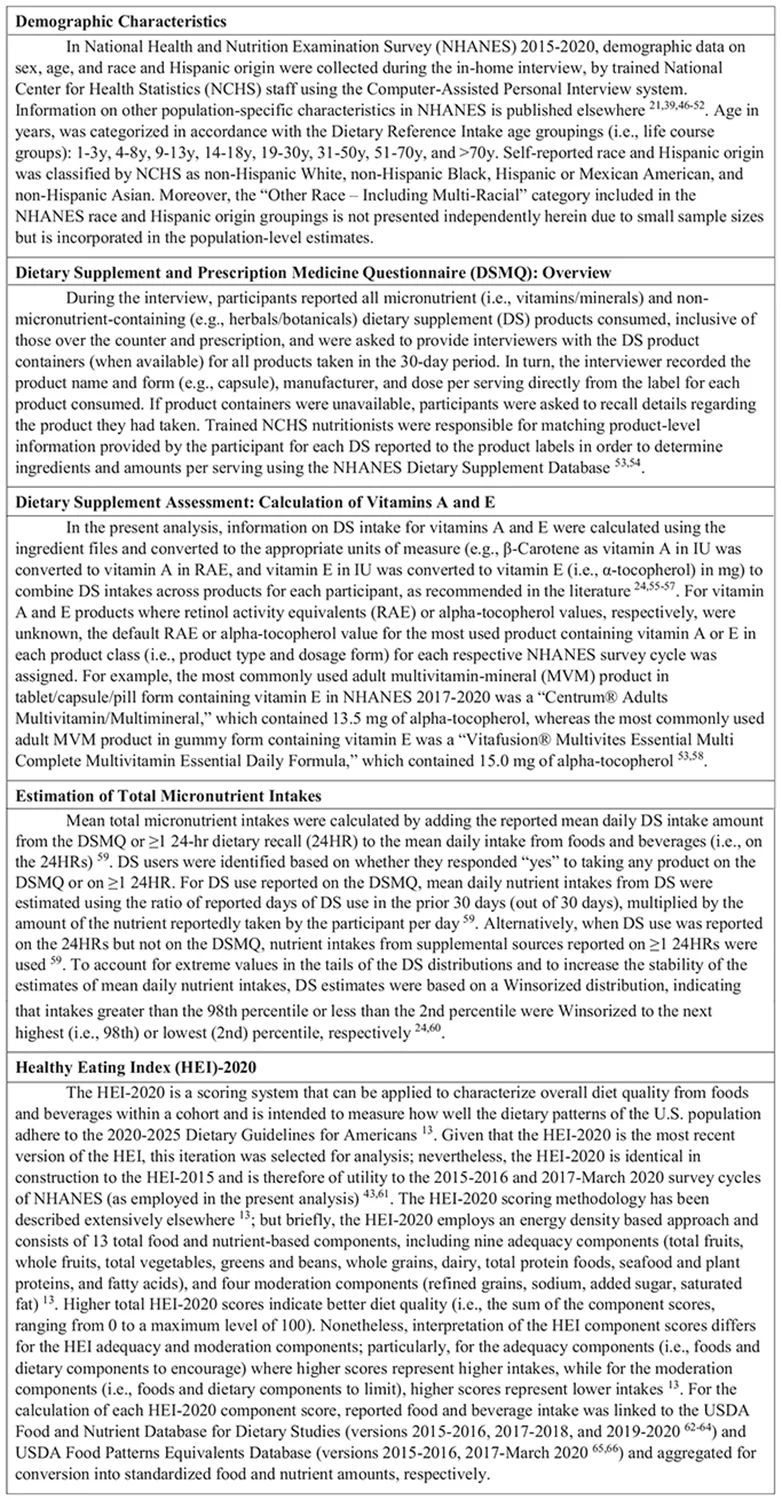
Supplemental methods on demographic characteristics, dietary supplement assessment, total micronutrient intakes, and dietary quality.

**Figure 2.**
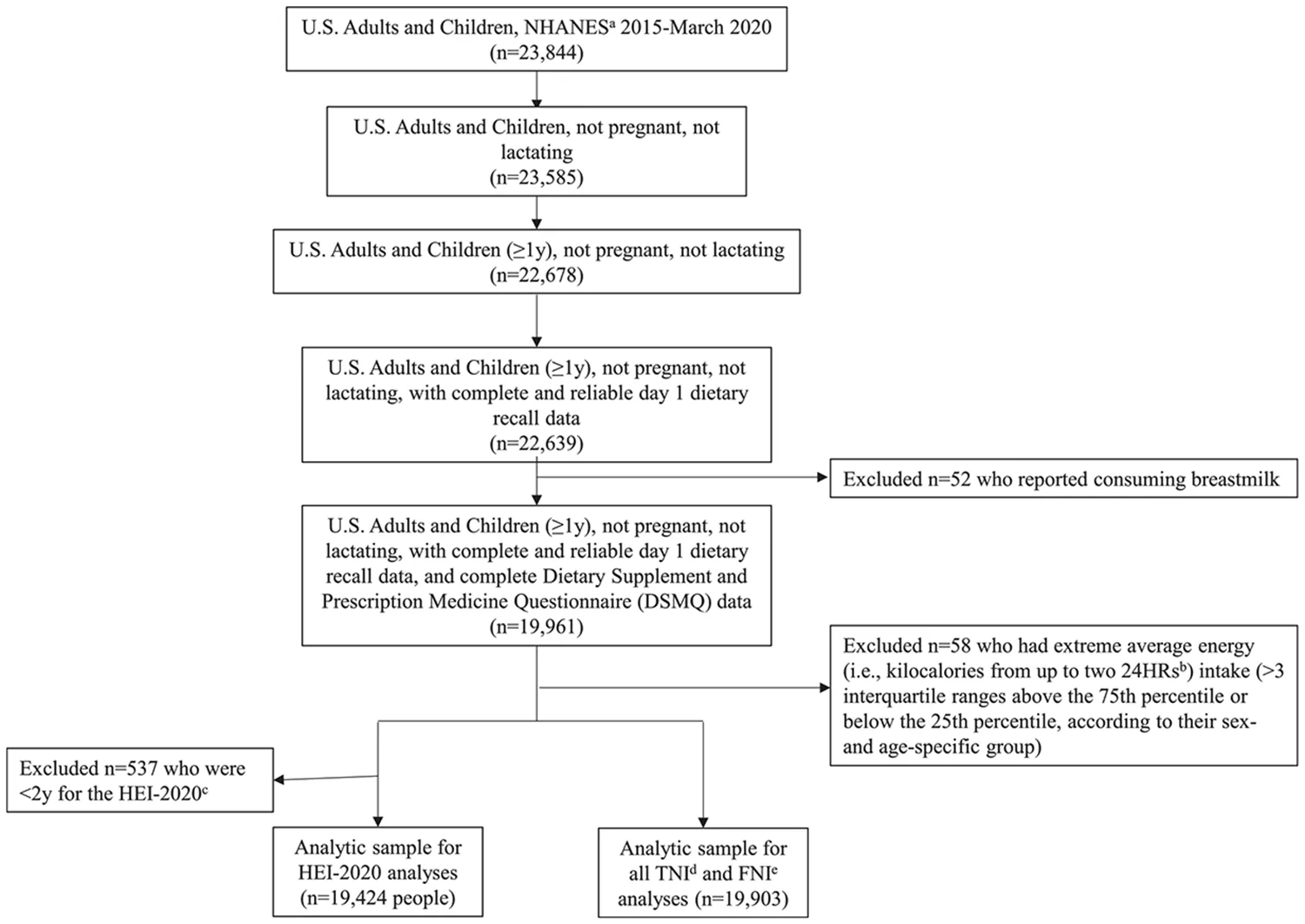
Participant flowchart outlining sample identification in the analysis of Total Nutrient Index, Food Nutrient Index, and Healthy Eating Index 2020 scores across the life course, using NHANES^a^ 2015-2020 data.^a^NHANES = National Health and Nutrition Examination Survey.^b^24HRs = 24-hour recalls.^c^HEI-2020 = Healthy Eating Index 2020.^d^TNI = Total Nutrient Index.^e^FNI = Food Nutrient Index.

Demographic information and dietary data were obtained for all participants in the 2015-2016 and 2017-March 2020 (pre-pandemic data) cycles of NHANES (detailed in Figure 1^13,21,24,39,41,41-62^, available at www.jandonline.org, and publicly available elsewhere^63,64^). Participants who were aged 1 year and older, were deemed reliable by interviewer assessment (ie, ≥1 complete and reliable 24-hour dietary recall [24HR]), had complete Dietary Supplement and Prescription Medicine Questionnaire (DSMQ) data, and were not pregnant or lactating were included in the analysis, as outlined in the participant flowchart (Figure 2). The analytic sample was further refined to exclude participants with extreme mean energy intake values (ie, kilocalories from up to 2 24HRs; >3 interquartile ranges above the 75th percentile or below the 25th percentile, according to their sex- and age-specific group^41,65,66^) by day, yielding a final analytic sample of n = 19 903 participants (ages 1 year and older).

### Dietary Assessment

Following the in-home interview, self-reported dietary intake data were collected via 2 interviewer-administered 24HRs, using the validated US Department of Agriculture (USDA) Automated Multiple Pass Method.^67,68^ The first 24HR was conducted in-person during the mobile examination center examination, whereas the second 24HR was conducted over the telephone ~3 to 10 days later^37^; both 24HRs collected information on all F&B and DS reportedly consumed from midnight to midnight the prior day. Proxy assistance and/or proxies were utilized for reporting intake for all participants younger than age 12 years, as described in detail elsewhere.^37,69^ In addition to the 24HRs, a DSMQ, in conjunction with an in-home DS product inventory, was administered in NHANES as an in-home interview module to compile information on participant DS use during the previous 30 days^70,71^; details on DSMQ data collection are in Figure 1 (available at www.jandonline.org).

The USDA Food and Nutrient Database for Dietary Studies (versions 2015-2016, 2017-2018, and 2019-2020^58-60^) and the USDA Food Patterns Equivalents Database (versions 2015-2016, 2017-March 2020^61,62^) were employed to convert all F&B reported in daily amounts of energy and NOFCs (eg, fiber). Nutrient values from DS were computed using the NHANES Dietary Supplement Database.^49,50^ However, micronutrient intakes from DS sources for vitamins A and E were not summarized in the publicly-available 2015-March 2020 NHANES DS data files (ie, DSMQ and 24HRs); further information on DS calculations for vitamins A and E are located in Figure 1 (available at www.jandonline.org), and additional details describing the NHANES DSMQ and 24HR protocols are outlined on the NHANES website.^69-71^

### TNI

The TNI is a micronutrient-based scoring metric that evaluates total micronutrient intakes from F&B and DS relative to the DRIs for adequacy. The FNI is an identical measure of micronutrient adherence but only from F&B and follows the same framework as the TNI. The initial development of the TNI and FNI indexes focused on 8 underconsumed micronutrients in the US population (ie, calcium, magnesium, potassium, choline, and vitamins A, C, D, and E).^23,24^ To score either the FNI (ie, from F&B only) or the TNI (ie, from F&B + DS), micronutrient intakes are expressed as a percentage of the age- and sex- specific Recommended Daily Allowance (RDA),^22,72^ when available, or the Adequate Intake^22,72^ when an RDA is unavailable. Each micronutrient component score is scored from 0 to 100 (with scores truncated at 100), and then averaged across all scores to yield the total TNI or FNI score, with equal weighting applied to each component.^23,24^ Comprehensive descriptions of the development and validation work for the TNI and FNI are published elsewhere.^23,24^ Details on the estimation of micronutrient intakes for the calculation of the TNI and FNI in the present analysis are in Figure 1 (available at www.jandonline.org).

**Figure 3.**
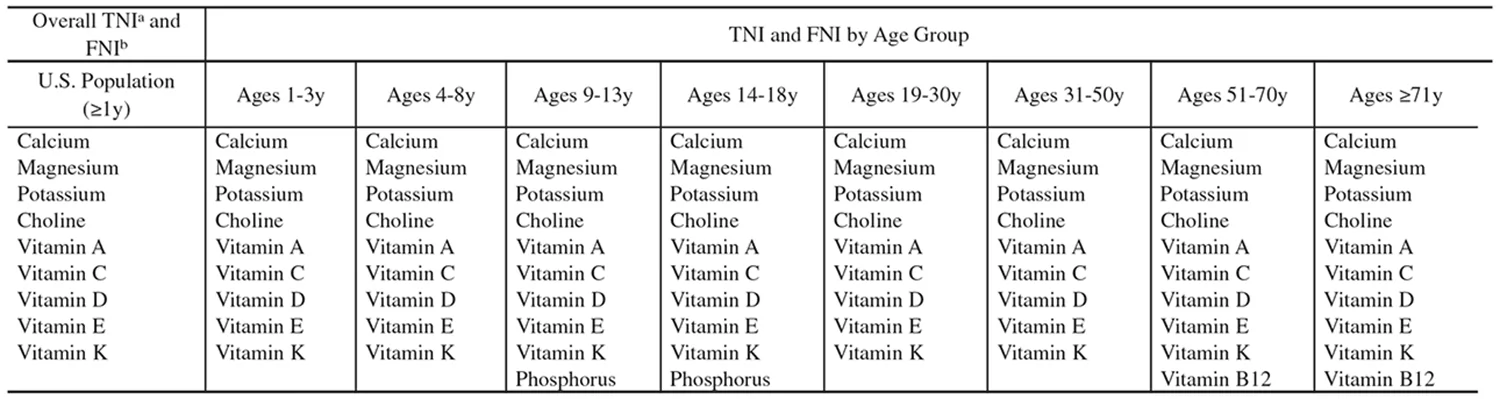
Total Nutrient Index and Food Nutrient Index Components, overall and by age group.^c a^TNI = Total Nutrient Index.^b^FNI = Food Nutrient Index.^c^Micronutrients included in this iteration of the TNI and FNI that were not components of the original TNI and FNI are phosphorus, vitamin B12 (age-group dependent) and vitamin K. The overall TNI and FNI reflect only micronutrients that are of public health relevance for the entirety of the US population aged 1 year and older.

The methodology presented in this analysis extends the TNI to incorporate a life course-based application for all ages (1 year or older). This iteration of the TNI employs 11 age group-dependent TNI components (ie, the nutrients included in the score vary with age), including calcium, choline, magnesium, phosphorus, potassium, and vitamins A, B12, C, D, E, and K, as detailed in Figure 3.^34^ These 11 micronutrients were identified as underconsumed either among the total US population (ie, calcium, magnesium, potassium, choline, and vitamins A, C, D, E, and K) and/or among specific age groups across the life course (ie, phosphorus in those aged 14 to 18 years and vitamin B12 in those aged 51 years and older).^34^ Although the TNI employs age group-dependent micronutrient components for each individual age group (eg, 10 nutrients are included in the TNI for adolescents aged 14 to 18 years), the overall TNI among the total US population reflects the 9 micronutrients that are of public health relevance for the entirety of the US population aged 1 year and older (Figure 3).

### Statistical Analysis

All statistical analyses were performed using SAS version 9.4.^73^ Appropriate statistical survey procedures (eg, *proc surveymeans, proc surveyfreq, proc surveyreg*) and Day 1 dietary sampling weights were used to account for the complex survey design of NHANES. SEs for all estimates were approximated using Taylor series linearization. Rao-Scott χ^2^ tests for categorical variables and analysis of variance for continuous variables (with incorporation and clustering for NHANES) were used to determine significant differences between demographic groups.

For each participant, HEI-2020 total and component scores, as well as TNI and FNI total scores and component scores (ie, for each nutrient), were estimated using the simple algorithm method.^23^ The HEI-2020 was estimated using the sum of the 2 days of the ratios, rather than any individual day, among those aged 2 years and older (n = 19 424) because the HEI-2020 is intended for use among those aged 2 years and older only. The scoring methodology for the HEI-2020 is described in Figure 1 (available at www.jandonline.org) and the SAS macros for the HEI simple algorithm method are publicly available on the NIH/NCI website.^74^ Ascending HEI-2020 “letter grade” categories (ranges of scores = 0 to 59 [F], 60 to 69 [D], 70 to 79 [C], and ≥80 [A/B]) utilized in this analysis were determined *a priori* and correspond with those outlined in Krebs-Smith and colleagues.^57^ Similarly, TNI scores were also grouped *a priori* into 3 TNI categories that correspond with the midrange of scores for the HEI-2020:^13^ <55 (low), 55-85 (middle), and >85 (high). Demographic characteristics, as well as mean TNI, FNI, and HEI-2020 total scores were examined across the 3 TNI categories (as described above); a Bonferroni-adjusted *P* value < .0167 was considered statistically significant.

## RESULTS

### US Population Aged 1 Year and Older

In NHANES 2015-2020, US adults and children (aged 1 year and older) were, on average, age 39 years, of non-Hispanic White descent (60%), and more likely to take a DS (55%) than not (Table 1). Across the TNI categories, mean FNI and HEI-2020 scores significantly increased with increasing category of TNI score. In addition to increases in micronutrient and dietary quality across the categories, notable differences in DS use were also noted according to category of TNI score; for example, 78% of those in the highest category (TNI >85) reported taking a DS, whereas only 25% in the lowest category (TNI <55) did so (Table 1). The average TNI (F&B + DS) score for US adults and children (aged 1 year and older) was 72 out of 100, whereas the average FNI (F&B only) score was 66 out of 100; scores for both indexes were predominantly driven by low intakes of vitamins A and D, and choline, compared with higher intakes of calcium, magnesium, and potassium (Table 2). Notably, mean component scores ranged from 78 to 79 out of 100 on the TNI for calcium, magnesium, and potassium, and from 53 to 68 out of 100 on the TNI for choline and vitamins A and D. Similar patterns were also observed among the FNI component scores. Total TNI and FNI scores by sex among the US population are presented in Table 3.

**Figure 4.**
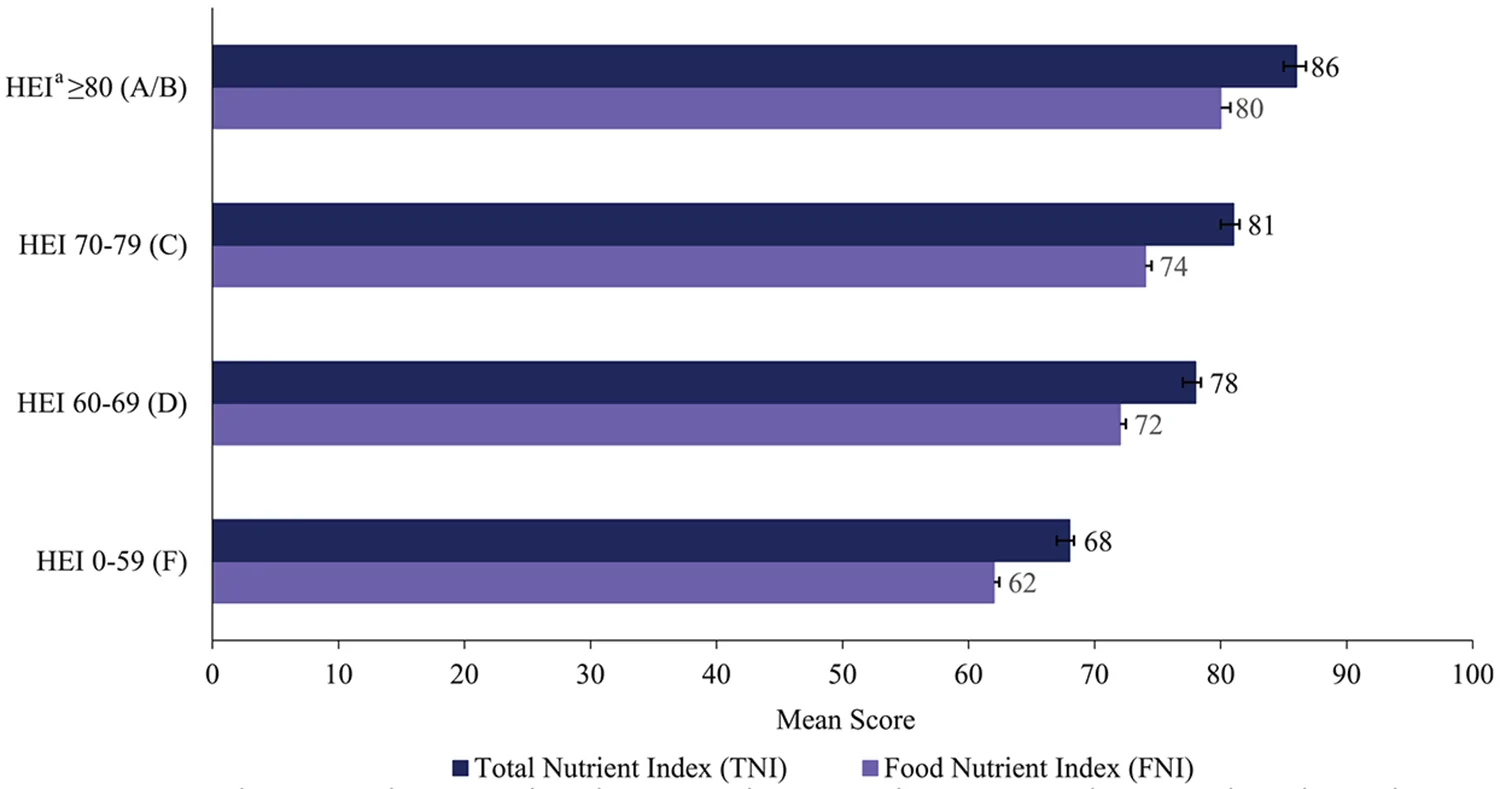
Mean Total Nutrient Index (TNI) and Food Nutrient Index (FNI) total scores among the US population by Heathy Eating Index 2020 score categories, National Health and Nutrition Examination Survey (NHANES) 2015-2020.^bcd a^HEI = Healthy Eating Index.^b^The analytic sample includes individuals ages 1 year and older (n = 19 903) who were not pregnant and/or lactating and with complete and reliable information for at least 1 24-hour dietary recall and the Dietary Supplement and Prescription Medicine Questionnaire. NHANES Day 1 dietary weights were utilized to obtain estimates for the US population. The error bars included in the bar chart reflect the SE of the estimated mean TNI or FNI score, respectively.^c^The micronutrient included in this iteration of the overall TNI and FNI that was not a component of the original TNI and FNI is vitamin K.^d^The HEI-2020 was designed to evaluate dietary intakes among the US population ages 2 years and older. Therefore, the analytic sample for the HEI-2020 is restricted to ages 2 years and older (n = 19 424). Ascending HEI-2020 score categories presented in this figure correspond with a graded approach: 0 to 59 (F), 60 to 69 (D), 70 to 79 (C), and ≥80 (A/B) to aid in interpretation of scores.

The mean HEI-2020 total score (52 out of 100) among the US population (aged 1 year and older) was generally low overall (Table 2). HEI-2020 adequacy component scores were markedly low for greens and beans (1.8 out of 5), whole grains (2.8 out of 10), seafood and plant protein (2.7 out of 5), and total fruits (2.3 out of 5), and for the moderation components, intakes of sodium (4.4 out of 10) and saturated fat (5.2 out of 10) particularly exceeded the recommended limit (Table 2). Similarly, when comparing TNI and HEI-2020 total scores, overall TNI scores (aged 1 year and older) increased with category of HEI-2020 score (eg, HEI-2020 ≥80: 86 out of 100 TNI; HEI-2020 0 to 59: 68 out of 100 TNI) (Figure 4).

### US Population Ages 1 Year and Older by Age Group

**Figure 5.**
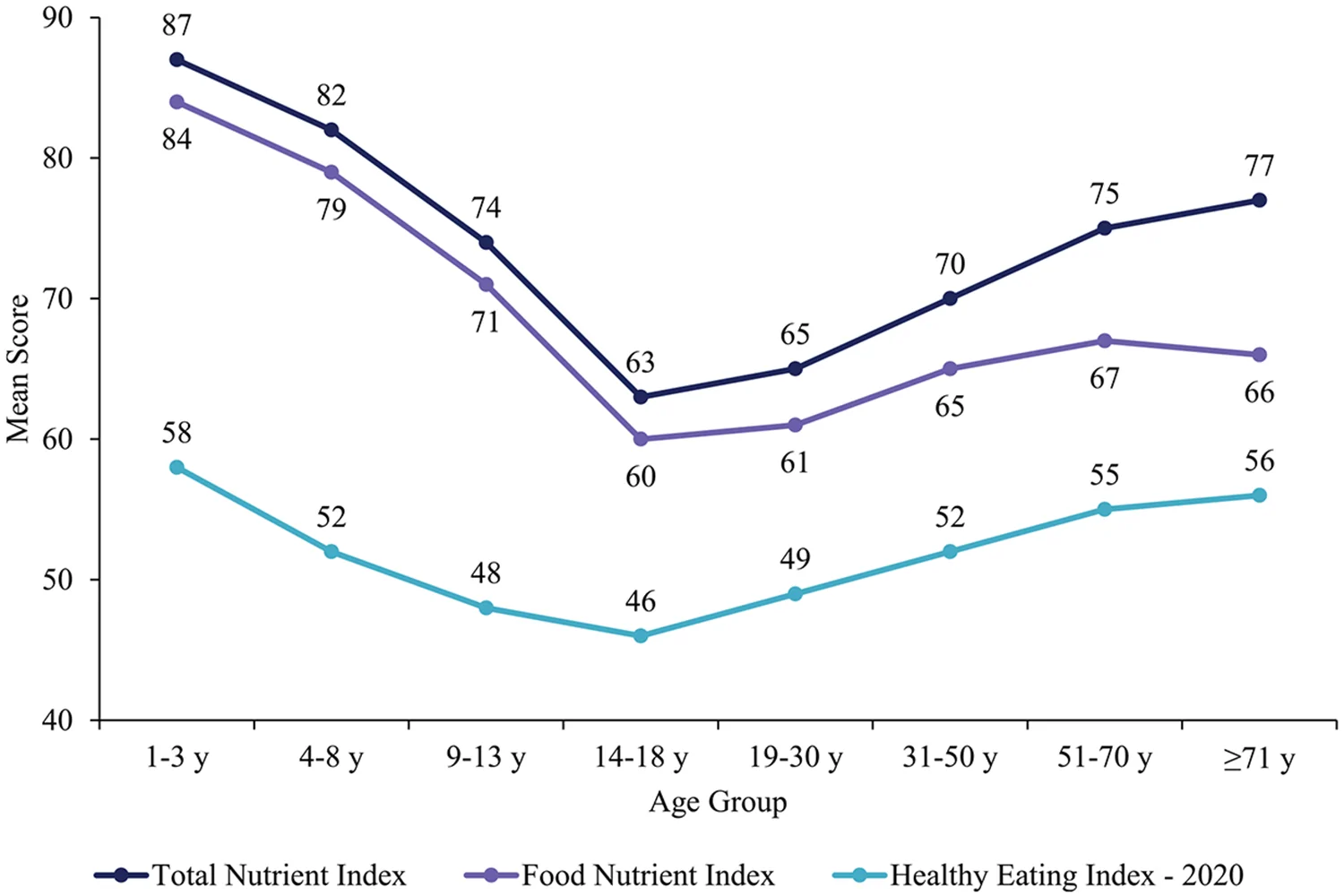
Mean Total Nutrient Index (TNI), Food Nutrient Index (FNI), and Healthy Eating Index 2020 (HEI-2020) total scores among the US population by age group, NHANES^a^ 2015-2020.^bcd a^NHANES = National Health and Nutrition Examination Survey.^b^The analytic sample includes individuals aged 1 year and older (n = 19 903) who were not pregnant and/or lactating and with complete and reliable information for at least 1 24-hour dietary recall and the Dietary Supplement and Prescription Medicine Questionnaire, by age group. NHANES Day 1 dietary weights were utilized to obtain estimates for the US population.^c^The TNI components include calcium, magnesium, potassium, phosphorus, choline, and vitamins A, C, D, E, and K, depending on the age group. Micronutrients included in this iteration of the age-group based TNI and FNI that were not components of the original TNI and FNI are phosphorus and vitamins B12 (age-group dependent) and K.^d^The HEI-2020 was designed to evaluate dietary intakes among the US population aged 2 years and older. Therefore, the overall analytic sample for the HEI-2020 is restricted to ages 2 years and older (n = 19 424). For the 1 to 3 years age group, the HEI-2020 analytic sample is reflective of those ages 2 to 3 years only (n = 881).

Across the life course, mean total TNI (range = 63 to 87 out of 100) and FNI (range = 60 to 84 out of 100) scores varied with age. Those in the youngest age groups (ie, 1 to 3 years and 4 to 8 years) had the highest mean scores on both the TNI (1 to 3 years: 87; 4 to 8 years: 82) and FNI (1 to 3 years: 84; 4 to 8 years: 79) of any age group (Figure 5). Thereafter, scores declined with age and were the lowest in adolescence (aged 14 to 18 years: TNI = 63, FNI = 60) and young adulthood (aged 19 to 30 y: TNI = 65, FNI = 61). Lastly, TNI and FNI scores then increased in middle-aged (31 to 50 years: TNI = 70, FNI = 65) and older (51 to 70 y: TNI = 75, FNI = 67; ages 71 years and older: TNI = 77, FNI = 66) adulthood. The TNI and FNI generally followed a similar decreasing trajectory during childhood and adolescence, with an average difference in TNI and FNI scores of ~3 points (Figure 5 and Table 4); however, in adulthood, the gap in average TNI and FNI scores widened with increasing age, and resulted in a maximum difference of approximately 11 points between scores (ie, TNI and FNI) within the older adult age group (Figure 5 and Table 5).

**Figure 6.**
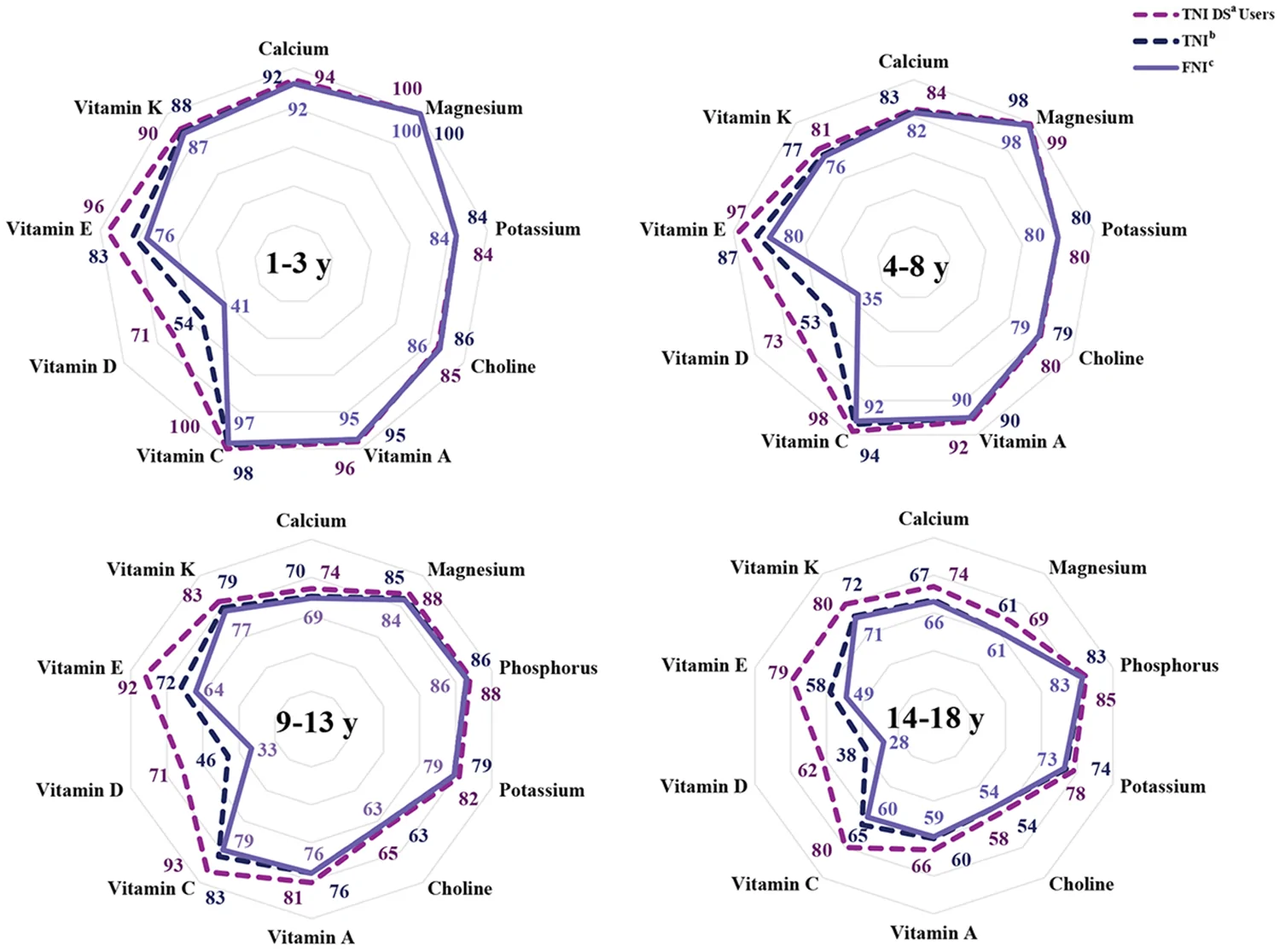
Mean Total Nutrient Index and Food Nutrient Index component scores among US children, by age group, NHANES^d^ 2015-2020^ef a^DS = dietary supplement.^b^TNI = Total Nutrient Index.^c^FNI = Food Nutrient Index.^d^NHANES = National Health and Nutrition Examination Survey.^e^The analytic sample includes individuals ages 1 to 18 years (n = 7143) who were not pregnant and/or lactating and with complete and reliable information for at least 1 24-hour dietary recall and the Dietary Supplement and Prescription Medicine Questionnaire, by age group. NHANES Day 1 dietary weights were utilized to obtain estimates for the US population.^f^Micronutrients included in this iteration of the age-group based TNI and FNI that were not components of the original TNI and FNI are phosphorus and vitamins B12 (age-group dependent) and K.

TNI component scores among younger children (aged 1 to 3 years and 4 to 8 years) were consistently high; however, children 4 to 8 years scored at least ~4 to 5 points lower on the TNI components for some nutrients (eg, calcium, choline, and vitamins A and K) and higher on others, such as vitamin E, than younger children (ages 1 to 3 years) (Figure 6 and Table 4). Among young adolescents ages 9 to 13 years, mean TNI component scores ranged from 46 to 86, and were highest among the TNI components for phosphorus (86 out of 100), magnesium (85 out of 100), and vitamin C (83 out of 100), and lowest among the TNI components for vitamin D (46 out of 100), choline (63 out of 100), and calcium (70 out of 100) (Figure 6 and Table 4). Among adolescents aged 14 to 18 years, mean component scores were the lowest of any other age group for most nutrients, with the lowest TNI component scores (all ≤62 out of 100) for choline, magnesium, and vitamins A, D, and E (Figure 6 and Table 4).

FNI component scores were only marginally different from TNI component scores among US children and adolescents (ages 1 to 18 years), and generally followed the same patterns as the TNI, with the sole exception being vitamin D (Figure 6 and Table 4).

Stratifying by DS use status indicated that the differences in TNI and FNI total scores are primarily driven by vitamins D and E for younger children (aged 1 to 3 years), and additionally by vitamin C for older children (aged 4 to 8 years); nonetheless, among adolescents (aged 9 to 13 years and ages 14 to 18 years), distinguishable differences in scores by DS user status were noted for all components (Figure 6).

**Figure 7.**
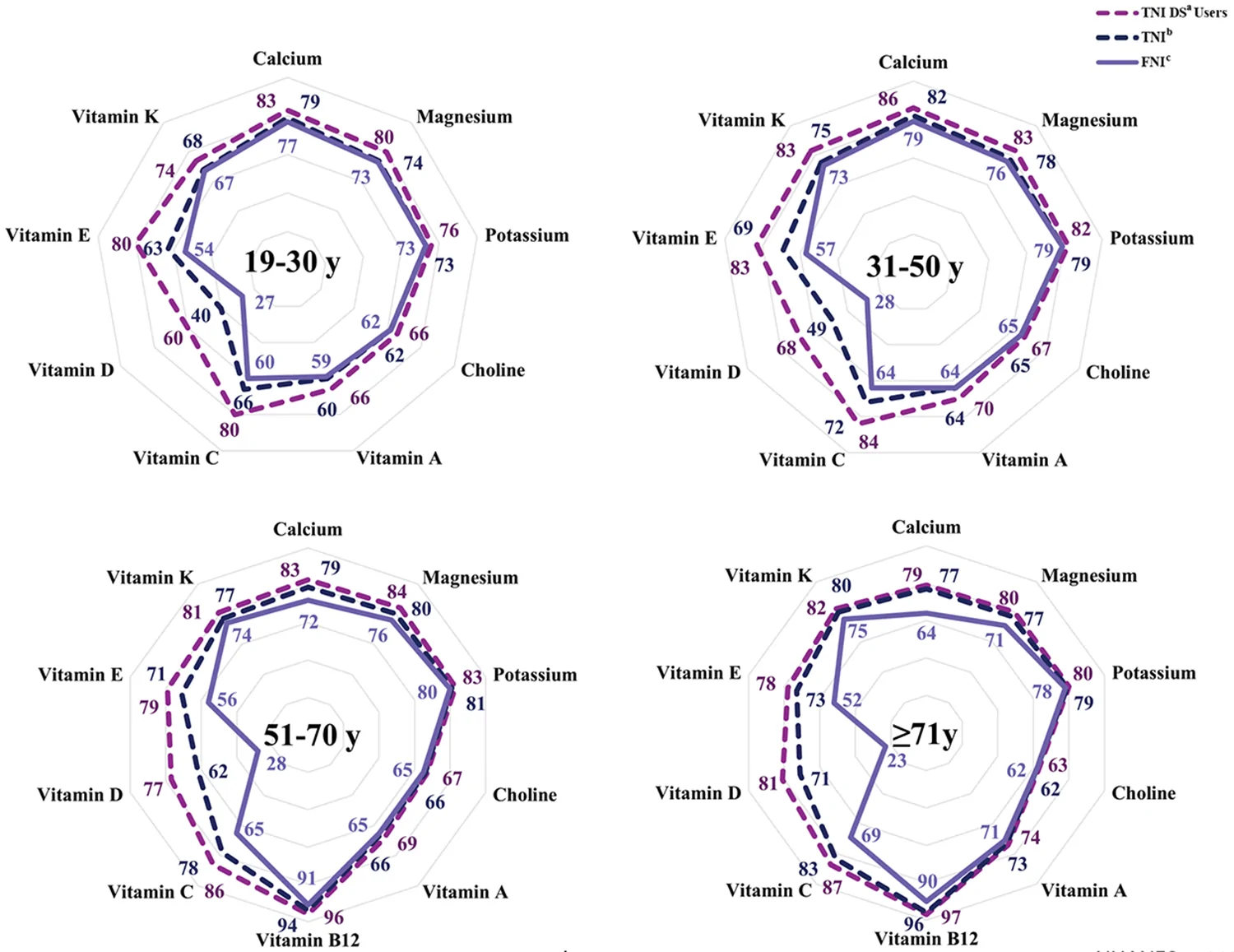
Mean Total Nutrient Index and Food Nutrient Index component scores among US adults, by age group, NHANES^d^ 2015-2020.^ef a^DS = dietary supplement.^b^TNI = Total Nutrient Index.^c^FNI = Food Nutrient Index.^d^NHANES = National Health and Nutrition Examination Survey.^e^The analytic sample includes individuals aged 19 years and older (n = 12 760) who were not pregnant and/or lactating and with complete and reliable information for at least 1 24-hour dietary recall and the Dietary Supplement and Prescription Medicine Questionnaire, by age group. NHANES Day 1 dietary weights were utilized to obtain estimates for the US population.^f^Micronutrients included in this iteration of the age group-based TNI and FNI that were not components of the original TNI and FNI are phosphorus and vitamins B12 (age-group dependent) and K.

**Figure 8.**
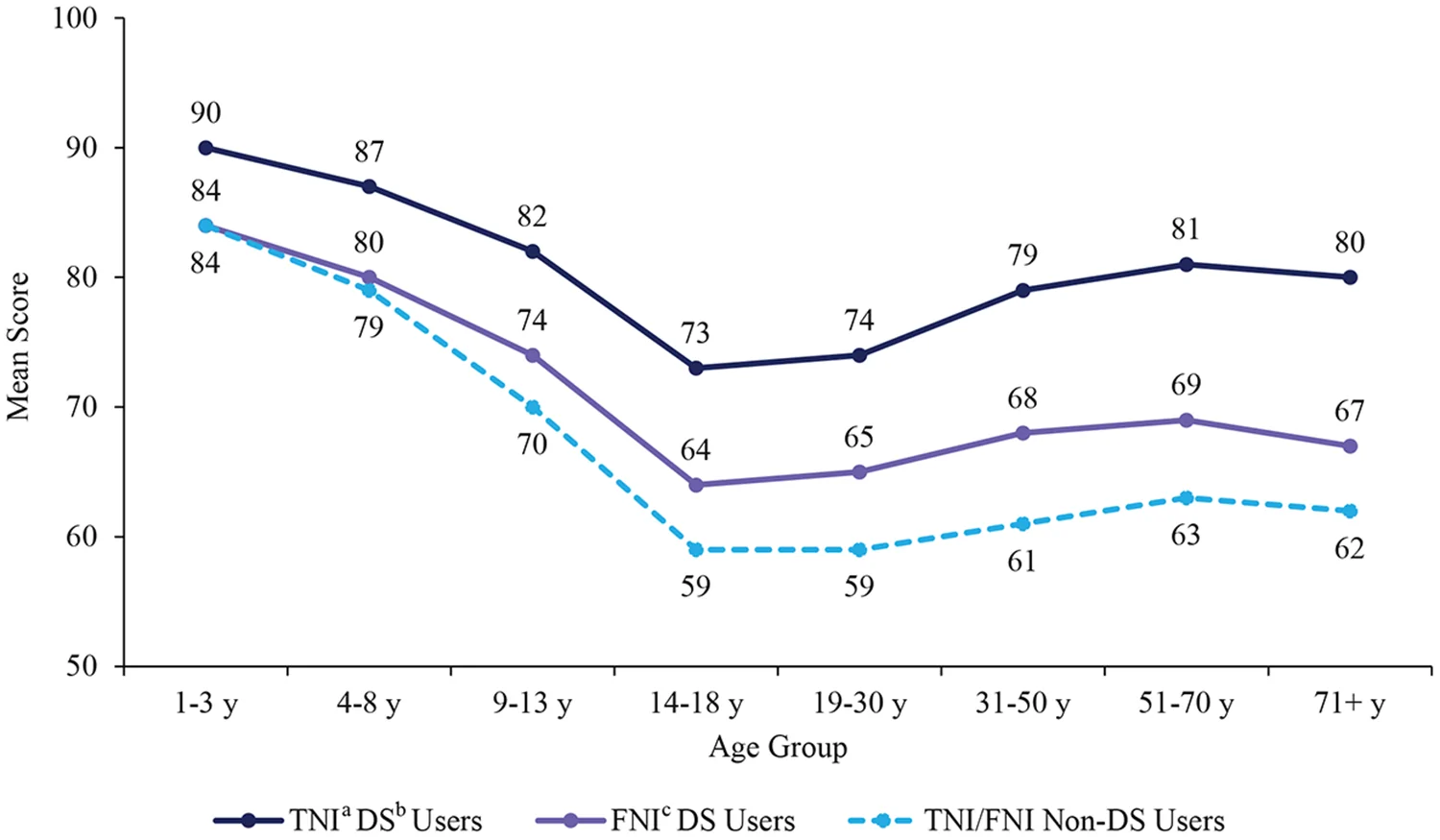
Mean Total Nutrient Index and Food Nutrient Index total and component scores among dietary supplement users and nonusers in the US population by age group, NHANES^d^ 2015-2020.^efg a^TNI = Total Nutrient Index.^b^DS = dietary supplement.^c^FNI = Food Nutrient Index.^d^NHANES = National Health and Nutrition Examination Survey.^e^The analytic sample includes individuals aged 1 year and older (n = 19 903) who were not pregnant and/or lactating and with complete and reliable information for at least 1 24-hour dietary recall and the Dietary Supplement and Prescription Medicine Questionnaire (DSMQ), by age group. NHANES Day 1 dietary weights were utilized to obtain estimates for the US population.^f^The TNI components include calcium, magnesium, potassium, phosphorus, choline, and vitamins A, C, D, E, and K, depending on the age group. Micronutrients included in this iteration of the age-group based TNI and FNI that were not components of the original TNI and FNI are phosphorus and vitamins B12 (age-group dependent) and K.^g^Dietary supplement use was classified based on whether the participant responded “yes” to “[taking] any vitamins, minerals, or other dietary supplements in the past month?” on the DSMQ or to “[taking] any vitamins, minerals, or other dietary supplements in the past 24 hours?” on at least 1 24-hour dietary recall.

For adults (ages 19 years and older) in NHANES, TNI component scores increased in a stepwise fashion with age. Scores across all adult age groups were consistently characterized by low intakes of choline, vitamins A, D, and E, and higher intakes of calcium, magnesium, potassium, and vitamins B12 and C, with some variations depending on age group (Figure 7 and Table 5). Middle-aged (ages 51 to 70 years) and older adults (ages 71 years and older) scored higher on several TNI components, including vitamins A, C, D, E, and K, than younger adults (ages 19 to 30 years and ages 31 to 50 years). The magnitude of the difference in TNI and FNI component scores was larger for nutrients commonly consumed in the form of DS, such as vitamins C, D, and E. For example, whereas total mean FNI scores (range = 61 to 69 out of 100) were somewhat lower than TNI scores among all adult subgroups (ages 19 years and older), FNI component scores for vitamins C, D, and E were substantially lower than TNI component scores among older adults (ages 71 years and older) by approximately 14, 48, and 21 points, respectively (Figure 7 and Table 5). Examining TNI and FNI scores among DS users revealed that DS use led to a much larger difference in mean TNI and FNI component scores for the nutrients that are highly consumed in the form of DS. For example, for vitamin D, the mean component score for the TNI among DS users exceeded the FNI score by 30 to 58 points, depending on age group (Figures 6 and 7). TNI and FNI total scores were also higher among DS users when compared with non-DS users, regardless of age group (Figure 8).

## DISCUSSION

Accurate, robust methodological approaches are warranted to assess how dietary exposures are associated with human health, as well as diet-related chronic disease. This extension of the TNI framework seeks to advance dietary exposure assessment methodology by addressing nutritional differences across the life course, dietary variability among population subgroups, and nutrient intakes from all sources. In the era of precision nutrition, addressing pervasive disease risk requires advanced precision in establishing essential nutrient requirements across the life span, considering effects of NOFCs on metabolic and physiological responses and the variability in contextual factors of diet,^75,76^ as well as the methodological approaches to measuring dietary exposures; yet none of these considerations are currently well understood.

US adults and children had a total TNI score of 72 out of 100, indicating low to moderate micronutrient quality of the diet. TNI scores fluctuated by age group in a *V* shape, with younger children (ages 1 to 8 years) and older adults (ages 71 years and older) exhibiting the highest levels of DRI adherence, and adolescents scoring lowest on the TNI, FNI, and HEI-2020 than any other life course group. These findings align with previous work^48,77-81^ suggesting that US adolescents and emerging adults are especially vulnerable to suboptimal nutrient and dietary patterns relative to other ages. Insufficient nutritional status and metabolic risk factors during adolescence and early adulthood uniquely positions this age group for significant nutritional risk, as they undergo a critical time point for development (eg, rapid growth and puberty)^34^ that can lead to a negative long-term diet and health trajectory.^1,48,82,83^ Adolescence and early adulthood are also periods of immense dietary transition, in which individuals undergo large shifts in contextual determinants (eg, caregiver involvement in dietary choices) that are key to understanding dietary variability and addressing drivers of poor dietary quality within these age groups.^48,81,84-87^

The TNI framework can be tailored to reflect micronutrients of interest for various populations or population subgroups, according to specific research question(s), and may be used in other clinical settings beyond research.^23^ This framework is especially useful for understanding the micronutrient density of nutrients ubiquitous to DS (eg, vitamin D)^21^ and/or among US population subgroups with notably high DS usage patterns, such as older adults.^43^ The TNI is a flexible framework that has the potential to be adapted to meet needs for nutrition assessment, screening, and monitoring.^23^ Moreover, other age-group dependent micronutrients have also been identified for certain sex- and age-specific life course groups that were not selected for inclusion in this iteration of the TNI. Most notably, the National Academies of Sciences, Engineering, and Medicine Review of the Special Supplemental Nutrition Program for Women, Infants, and Children Food Package report identified iron, copper, zinc, and folate, thiamin, riboflavin, and vitamin B6 as additional shortfall nutrients relevant to certain population subgroups (eg, pregnant women, lactating women, and breastfed infants).^20^ Although these nutrients are highly pertinent to some sex- and age-specific groups, the focus of this TNI is on the general US population across the life course, as opposed to specifically targeting women, infants, and children (ie, the nutrients in the Special Supplemental Nutrition Program for Women, Infants, and Children food package).

Correspondingly, iron, folate, and vitamins B6 and B12 have been previously noted as nutrients of public health relevance among female adolescents aged 14 to 18 years^35,77^; however, given that these micronutrients are not applicable to the entirety of the life course group (d, both sexes), and may exert undue influence on group-level TNI estimates for this age group (ie, adolescents aged 14 to 18 years), our investigative team chose not to include these nutrients in the final list of micronutrients included among those aged 14 to 18 years. Intakes of these nutrients among adolescents are well-described elsewhere.^25,77,88,89^

In addition to the selection of age-group dependent micronutrients, this iteration of the TNI sought to evaluate micronutrient quality among the nonpregnant, nonlactating US population 1 year and older. Adequate sample sizes were not available in NHANES 2015-2020 to explore TNI total and component scores among US infants and pregnant and lactating women in our current analysis; but future explorations of the TNI in other cohorts with sufficient sample size among these population subgroups is salient.

More work is needed to not only extend the TNI framework to accommodate other population subgroups, but also to expand upon the TNI validation work and statistical modeling techniques used in this application of the TNI. At this time, the TNI scoring system has been validated among the US adult population, predominantly due to the limitations surrounding nutritional biomarker availability in NHANES and the ability to validate the index among US children using this dataset; hence, our investigative team plans to explore the potential for TNI validation among US children in other cohort(s).^90^ This study provides a descriptive analysis of micronutrient quality among the US population across the life course; while additional efforts to establish the relative validity and reliability of the index are needed, the TNI (range = 63 to 87 out of 100) was consistent with and followed a similar trajectory across age groups to that of the HEI-2020 (range = 46 to 58 out of 100).^13^

### Strengths and Limitations

Several statistical methods can be used to calculate dietary index scores (eg, simple algorithm, population ratio, or multivariate).^23,91^ Calculation of the index requires 3 distinct steps: first, dietary intake is assessed, which may be done through the use of different types of diet assessment tools (eg, food frequency questionnaire, 24HR, or food diary); second, the dietary data are summarized, which is dependent on the diet assessment tool and the methods used to derive typical dietary intakes (eg, using recipes and responses linked to typical intakes on the food frequency questionnaire, using 1 day from a 24HR, using the simple average of days from food diary, or using measurement error correction methods to estimate “usual intakes”); finally, the algorithm that defines the index is applied to calculate the index score. Although the final step is consistent across methods, both the choice of dietary assessment tool and the way in which dietary data are summarized can impact estimates of index scores and population-level conclusions. A strength of this analysis is that the dietary data in NHANES are collected using the USDA Automated Multiple-Pass Method method^67,68^ for 24HRs and the in-home DS inventory. However, all self-reported dietary^92^ and DS data are prone to some degree of measurement error, and the measurement error structure of DS reporting is unknown.^56,93^ In this analysis, we used the average of 24HR data in a simple algorithm method to estimate mean TNI, FNI, and HEI-2020 scores. Given that this analysis was restricted to analysis of means, resultant findings are expected to yield similar mean estimates to that of more complex methods to estimate usual intake from the 24HR data. Nevertheless, use of a more complex method to adjust for measurement error would be necessary to provide more precise estimates of the distribution of TNI and FNI scores in the US population.

The cross-sectional nature of the NHANES survey limits the ability to infer causality or temporality in the present analysis. In addition, challenges and limitations exist with the use of food and nutrient composition databases,^94-96^ such as the USDA Food and Nutrient Database for Dietary Studies,^58-60^ to derive micronutrient intake values, due to concerns surrounding data quality (eg, timeliness of data collection/analysis, representativeness of sampling due to food availability, regionality, and seasonality), food component variability (eg, inherent, environmental, and processing/preparation techniques), and database processing decisions (eg, merging of data sources and food component naming).^95^ Similar challenges also arise with DS assessment and DS composition databases because current methods require reliance on label-declared product values, which may deviate from the actual contents of the product inside the DS container.^97^ However, food and nutrient composition data are an essential component of nutrient intake assessment and the development of food- and nutrient-based dietary guidance^96^; utilization of nationally representative population-based data and the corresponding food and nutrient composition databases in this study allowed for broad applicability of the findings with the TNI. Lastly, the DRI standards for micronutrient adequacy included in the TNI framework are periodically updated by the National Academies of Sciences, Engineering, and Medicine (eg, most recently potassium in 2019^98^). In addition, the strength of the scientific evidence in which the DRIs are established varies by nutrient (eg, some nutrients with an established RDA vs an Adequate Intake), especially among certain population subgroups, and therefore inclusion of the DRIs in the TNI framework should be considered in light of this caveat. Finally, DRI standards may become available for other micronutrients or dietary constituents of public health significance that may warrant inclusion in future iterations of the TNI.

## CONCLUSIONS

Understanding the micronutrient quality of dietary exposures across the life span and extending advanced techniques for comprehensive exposure classification are critical for achieving accurate, population-based nutrient recommendations in the era of precision nutrition. Micronutrient patterns and their quality vary by age group among the US population, with low DRI adherence for several micronutrients across population subgroups, even with the inclusion of contributions from DS. Thus, a significant need exists for tailored, precision nutrition approaches that can accurately identify how dietary exposures differentially influence all ages across the life course, and in turn, inform public health recommendations to reduce risk of diet-related chronic disease. Future research endeavors investigating how these differences in micronutrient quality relate to risk of morbidity and mortality in different populations and with different dietary assessment tools are merited.

## Supplementary Material

Supplemental File Labeled at Figure 1

Figure 1 is available at www.jandonline.org

## Figures and Tables

**Table 1.** Demographic and lifestyle characteristics of the US population, National Health and Nutrition Examination Survey (NHANES) 2015-2020, overall and by category of Total Nutrient Index (TNI) score^a^

Characteristic	US Population Aged 1 y and Older, Overall and by TNI Score Category								
	n	Total (n = 19 903)	n	TNI <55^b^ (n = 4261)	n	TNI 55-85^b^ (n = 9484)	n	TNI >85^b^ (n = 6158)	*P* value^c^
		*mean (SE)*		*mean (SE)*		*mean (SE)*		*mean (SE)*	
**TNI score**	19903	71.6 (0.4)	4261	41.3 (0.3)	9484	70.2 (0.1)	6158	91.7 (0.1)	< .0001
**FNI**^d^ **score**	19903	65.5 (0.4)	4261	39.7 (0.3)	9484	64.4 (0.2)	6158	82.7 (0.2)	< .0001
**HEI-2020**^e^ **score**^f^	19424	52.1 (0.4)	4249	44.2 (0.4)	9313	51.2 (0.3)	5862	58.0 (0.4)	< .0001
**Sex**		*% (SE)*		*% (SE)*		*% (SE)*		*% (SE)*	.2520
Male	9922	49.5 (0.6)	2142	51.3 (1.0)	4703	48.7 (1.0)	3081	50.6 (1.0)	
Female	9981	50.5 (0.6)	2119	48.7 (1.0)	4781	51.3 (1.0)	3077	49.4 (1.0)	
		*mean (SE)*		*mean (SE)*		*mean (SE)*		*mean (SE)*	
**Age (y)**	19903	39.1 (0.4)	4261	36.9 (0.5)	9484	39.1 (0.5)	6158	40.4 (0.7)	< .0001
		*% (SE)*		*% (SE)*		*% (SE)*		*% (SE)*	
**Race and Hispanic origin**									< .0001
Non-Hispanic White	6744	60.3 (2.2)	1169	52.2 (2.7)	3114	59.7 (2.3)	2461	66.0 (2.2)	
Hispanic or Mexican American	5174	17.8 (1.6)	1169	20.9 (1.9)	2534	18.3 (1.7)	1471	15.1 (1.5)	
Non-Hispanic Black	4911	11.8 (1.3)	1347	17.5 (1.8)	2384	12.0 (1.3)	1180	8.2 (1.0)	
Non-Hispanic Asian	1906	5.5 (0.7)	345	5.0 (0.7)	906	5.7 (0.8)	655	5.6 (0.8)	
Other Race/Hispanic origin^g^	1168	4.5 (0.3)	231	4.3 (0.5)	546	4.3 (0.3)	391	5.1 (0.5)	
**Overall dietary supplement use** ^h^									< .0001
Yes	9767	55.2 (0.8)	1000	25.0 (1.0)	4454	52.1 (0.9)	4313	77.8 (1.0)	
No	10136	44.8 (0.8)	3261	75.0 (1.0)	5030	47.9 (0.9)	1845	22.2 (1.0)	

a The analytic sample includes individuals aged 1 year or older (n = 19 903) who were not pregnant and/or lactating and with complete and reliable information for at least 1 24-hour dietary recall and the Dietary Supplement and Prescription Medicine Questionnaire. NHANES Day 1 dietary weights were utilized to weight to the US population; estimates account for the complex sampling scheme of NHANES and use Taylor linearization for SEs. Although prevalence estimates reported in this table are nationally representative (ie, weighted estimates), the sample sizes reflect the analytic sample.

b TNI categories (<55 [low], 55-85 [middle], and >85 [high]) were determined *a priori*.

c Analyses accounted for the complex survey design of NHANES. *P* values represent Rao-Scott χ^2^ tests for categorical variables and Wald *F* tests with Taylor series linearization for continuous variables. For continuous variables, *P* values presented in the table reflect the significance level for all estimates in a row, unless otherwise specified. A Bonferroni-corrected *P* < .0167 was considered statistically significant.

d FNI = Food Nutrient Index.

e HEI-2020 = Healthy Eating Index-2020.

f HEI-2020 was designed to evaluate dietary intakes among the US population ages 2 years and older. Therefore, the analytic sample for the HEI-2020 is restricted to participants aged 2 years and older (n = 19 424).

g The “Other Race/Hispanic origin” category includes multiracial participants.

h Dietary supplement use was classified based on whether the participant responded “yes” to “[taking] any vitamins, minerals, or other dietary supplements in the past month?” on the DSMQ or to “[taking] any vitamins, minerals, or other dietary supplements in the past 24 hours?” on at least 1 24-hour dietary recall.

**Table 2.** Mean Food Nutrient Index (FNI), Total Nutrient Index (TNI), and Healthy Eating Index (HEI)-2020 total and component scores among the US population, National Health and Nutrition Examination Survey (NHANES) 2015-2020^ab^

	US Population Aged 1 y and Older	
FNI/TNI component	FNI (n = 19 903)	TNI (n = 19 903)
	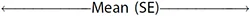	
Calcium	74.8 (0.5)	79.0 (0.5)
Magnesium	76.7 (0.4)	79.1 (0.5)
Potassium	78.1 (0.4)	78.3 (0.4)
Choline	65.0 (0.3)	65.2 (0.3)
Vitamin A	67.4 (0.6)	68.0 (0.6)
Vitamin C	67.8 (0.6)	76.2 (0.6)
Vitamin D	28.7 (0.4)	52.7 (0.7)
Vitamin E	58.2 (0.4)	70.1 (0.5)
Vitamin K	73.2 (0.5)	75.6 (0.5)
**Total FNI/TNI score**	**65.5 (0.4)**	**71.6 (0.4)**
HEI-2020 components^c^	HEI-2020 (n = 19 424)	
Adequacy		
Total Fruits (5)	2.3 (0.0)	
Whole Fruits (5)	2.5 (0.0)	
Total Vegetables (5)	3.0 (0.0)	
Greens and Beans (5)	1.8 (0.0)	
Whole Grains (10)	2.8 (0.1)	
Dairy (10)	5.4 (0.1)	
Total Protein Foods (5)	4.3 (0.0)	
Seafood and Plant Protein (5)	2.7 (0.0)	
Fatty Acids (10)	4.6 (0.1)	
Moderation		
Refined Grains (10)	6.1 (0.1)	
Sodium (10)	4.4 (0.1)	
Added Sugars (10)	7.0 (0.1)	
Saturated Fats (10)	5.2 (0.1)	
**Total HEI-2020 score**	**52.1 (0.4)**	

a The analytic sample includes individuals aged 1 year and older (n = 19 903) who were not pregnant and/or lactating and with complete and reliable information for at least 1 24-hr dietary recall and the Dietary Supplement and Prescription Medicine Questionnaire. NHANES Day 1 dietary weights were utilized to weight to the US population; estimates account for the complex sampling scheme of NHANES and use Taylor linearization for SEs Although prevalence estimates reported in this table are nationally representative (ie, weighted estimates), the sample sizes reflect the analytic sample.

b The micronutrient included in this iteration of the overall TNI and FNI that was not a component of the original TNI and FNI is vitamin K.

c HEI-2020 was designed to evaluate dietary intakes among the US population aged 2 years and older. Therefore, the analytic sample for the HEI-2020 is restricted to ages 2 years and older (n = 19 424). For the HEI-2020 adequacy components, higher scores represent higher intakes, whereas for the moderation components, higher scores represent lower intakes. Maximum values for each subcategory are listed in parentheses.

**Table 3.** Mean Total Nutrient Index (TNI) and Food Nutrient Index (FNI) total and component scores among the US population by sex, National Health and Nutrition Examination Survey (NHANES) 2015-2020^abc^

	US Population Aged 1 y and Older	
	Male (n = 9922)	Female (n = 9 981)
	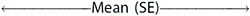	
TNI component		
Calcium	82.5 (0.5)	75.5 (0.6)
Magnesium	78.5 (0.5)	79.7 (0.5)
Potassium	77.5 (0.4)	79.2 (0.5)
Choline	66.3 (0.4)	64.1 (0.5)
Vitamin A	65.7 (0.7)	70.3 (0.8)
Vitamin C	74.4 (0.7)	78.1 (0.7)
Vitamin D	51.6 (0.7)	53.7 (0.9)
Vitamin E	71.8 (0.6)	68.5 (0.5)
Vitamin K	72.9 (0.5)	78.2 (0.6)
**Total TNI score**	**71.2 (0.4)**	**71.9 (0.5)**
FNI component		
Calcium	80.3 (0.5)	69.5 (0.6)
Magnesium	76.1 (0.4)	77.2 (0.5)
Potassium	77.2 (0.4)	78.9 (0.5)
Choline	66.2 (0.4)	63.9 (0.5)
Vitamin A	65.2 (0.7)	69.6 (0.8)
Vitamin C	66.3 (0.6)	69.2 (0.8)
Vitamin D	31.6 (0.5)	25.9 (0.4)
Vitamin E	62.0 (0.5)	54.4 (0.5)
Vitamin K	70.6 (0.6)	75.8 (0.6)
**Total FNI score**	**66.2 (0.4)**	**64.9 (0.4)**

a The analytic sample includes individuals aged 1 year and older (N = 19 903) who were not pregnant and/or lactating and with complete and reliable information for at least 1 24-hour dietary recall and the Dietary Supplement and Prescription Medicine Questionnaire. NHANES Day 1 dietary weights were utilized to obtain estimates for the US population.

b The micronutrient included in this iteration of the overall TNI and FNI that was not a component of the original TNI and FNI is vitamin K.

c Although mean estimates reported in this table are nationally representative (ie, weighted estimates), the sample sizes reflect the analytic sample.

**Table 4.** Mean Total Nutrient Index (TNI) and Food Nutrient Index (FNI) total and component scores among US children by age- and sex- specific life course group, National Health and Nutrition Education Survey (NHANES) 2015-2020^abc^

TNI component	Ages 1-3 y		Ages 4-8 y		Ages 9-13 y		Ages 14-18 y	
	Males (n = 707)	Females (n = 653)	Males (n = 977)	Females (n = 942)	Males (n = 982)	Females (n = 1024)	Males (n = 957)	Females (n = 901)
	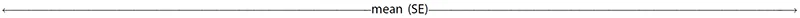							
Calcium	92.6 (0.9)	92.4 (0.9)	85.6 (0.8)	79.5 (0.8)	71.7 (1.2)	67.8 (1.0)	73.0 (1.2)	61.4 (1.8)
Magnesium	99.5 (0.1)	99.7 (0.1)	98.9 (0.2)	98.0 (0.3)	85.1 (0.9)	83.9 (0.7)	63.4 (1.0)	59.4 (1.6)
Phosphorus	–	–	–	–	87.1 (0.8)	84.2 (0.6)	88.7 (0.8)	76.9 (1.7)
Potassium	84.5 (1.1)	83.7 (0.9)	82.1 (0.8)	77.7 (0.8)	77.9 (1.1)	79.9 (0.8)	72.9 (1.1)	74.1 (1.6)
Choline	87.1 (0.9)	84.5 (1.1)	80.7 (0.8)	77.1 (1.0)	64.5 (1.1)	60.6 (0.9)	54.6 (1.3)	53.0 (1.4)
Vitamin A	95.0 (0.6)	94.6 (0.9)	91.2 (0.8)	89.4 (0.9)	77.5 (1.3)	75.4 (1.2)	58.5 (1.5)	61.1 (1.5)
Vitamin C	97.7 (0.5)	98.1 (0.5)	93.3 (1.1)	95.5 (0.6)	82.9 (1.8)	83.0 (1.5)	62.9 (1.6)	67.1 (2.0)
Vitamin D	55.4 (1.4)	53.5 (1.8)	53.7 (1.6)	51.6 (1.7)	46.2 (2.1)	45.4 (1.7)	40.1 (1.6)	36.7 (1.8)
Vitamin E	83.0 (1.1)	83.2 (1.4)	88.2 (0.9)	85.7 (1.0)	72.6 (1.7)	71.4 (1.3)	59.6 (1.3)	55.5 (1.4)
Vitamin K	89.1 (1.1)	86.6 (1.4)	78.4 (1.2)	76.5 (1.0)	77.8 (1.1)	79.2 (1.3)	72.5 (1.3)	71.7 (1.7)
**Total TNI score**	**87.1 (0.6)**	**86.3 (0.6)**	**83.6 (0.5)**	**81.2 (0.5)**	**74.3 (0.9)**	**73.1 (0.6)**	**64.6 (0.9)**	**61.7 (1.2)**
FNI component								
Calcium	92.4 (0.9)	92.3 (0.9)	85.3 (0.8)	79.0 (0.8)	71.0 (1.3)	67.3 (1.0)	72.4 (1.2)	59.7 (1.9)
Magnesium	99.5 (0.1)	99.7 (0.1)	98.9 (0.2)	98.0 (0.3)	85.0 (0.9)	83.8 (0.7)	63.0 (1.0)	58.1 (1.6)
Phosphorus	–	–	–	–	87.0 (0.8)	84.2 (0.6)	88.7 (0.8)	76.8 (1.7)
Potassium	84.5 (1.1)	83.7 (0.9)	82.0 (0.8)	77.7 (0.8)	77.9 (1.1)	79.9 (0.8)	72.9 (1.1)	74.0 (1.6)
Choline	87.0 (0.9)	84.5 (1.1)	80.6 (0.8)	77.0 (1.0)	64.4 (1.1)	60.6 (0.9)	54.5 (1.3)	52.9 (1.4)
Vitamin A	94.9 (0.6)	94.5 (0.9)	91.1 (0.8)	89.1 (0.9)	77.3 (1.3)	75.2 (1.2)	58.3 (1.5)	60.6 (1.5)
Vitamin C	96.4 (0.7)	97.5 (0.7)	91.2 (1.3)	93.1 (0.8)	78.5 (2.1)	79.4 (1.6)	58.7 (1.7)	60.5 (2.3)
Vitamin D	41.6 (1.0)	39.5 (1.2)	37.4 (0.8)	32.6 (0.7)	34.3 (1.0)	31.5 (0.9)	33.7 (1.4)	23.2 (0.9)
Vitamin E	75.9 (1.3)	75.3 (1.3)	82.7 (1.1)	76.2 (1.0)	64.7 (1.1)	63.0 (1.0)	53.7 (1.2)	45.1 (1.6)
Vitamin K	88.7 (1.1)	86.3 (1.4)	77.1 (1.1)	75.0 (1.0)	76.1 (1.2)	78.1 (1.3)	71.6 (1.2)	69.6 (2.0)
**Total FNI score**	**84.6 (0.6)**	**83.7 (0.5)**	**80.7 (0.5)**	**77.5 (0.5)**	**71.6 (0.9)**	**70.3 (0.6)**	**62.7 (0.9)**	**58.1 (1.4)**

a The analytic sample includes individuals ages 1 to 18 years (N = 7143) who were not pregnant and/or lactating and with complete and reliable information for at least 1 24-hour dietary recall and the Dietary Supplement and Prescription Medicine Questionnaire. NHANES Day 1 dietary weights were utilized to obtain estimates for the US population.

b Micronutrients included in this iteration of the TNI and FNI that were not components of the original TNI and FNI are phosphorus (age-group dependent) and K.

c Although mean estimates reported in this table are nationally representative (ie, weighted estimates), the sample sizes reflect the analytic sample.

**Table 5.** Mean Total Nutrient Index (TNI) and Food Nutrient Index (FNI) total and component scores among US Adults by age- and sex-specific life course group, National Health and Nutrition Examination Survey (NHANES) 2015-2020^abc^

TNI component	Ages 19-30 y		Ages 31-50 y		Ages 51-70 y		Ages ≥71 y	
	Men (n = 1185)	Women (n = 1173)	Men (n = 1943)	Women (n = 2057)	Men (n = 2206)	Women (n = 2293)	Men (n = 965)	Women (n = 938)
	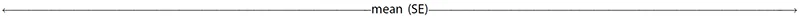							
Calcium	82.5 (0.9)	75.9 (1.1)	85.2 (0.9)	79.4 (0.9)	85.1 (0.8)	73.7 (1.2)	76.7 (1.2)	76.6 (0.9)
Magnesium	72.3 (1.0)	76.0 (1.2)	77.3 (0.8)	78.9 (0.8)	79.1 (0.8)	80.2 (0.9)	74.6 (1.0)	79.4 (0.7)
Potassium	70.5 (1.0)	76.0 (1.0)	78.4 (0.8)	79.3 (0.8)	80.1 (0.7)	81.3 (0.8)	77.7 (0.9)	80.3 (1.0)
Choline	62.1 (1.1)	62.2 (1.0)	66.5 (0.8)	64.2 (0.9)	67.1 (0.9)	64.3 (0.8)	62.2 (1.1)	61.4 (0.9)
Vitamin A	56.4 (1.5)	63.5 (1.2)	60.9 (1.2)	67.3 (1.4)	62.9 (1.0)	68.6 (1.2)	69.9 (1.2)	74.8 (1.1)
Vitamin B12	–	–	–	–	95.6 (0.5)	92.6 (0.6)	96.6 (0.5)	94.9 (0.6)
Vitamin C	62.7 (1.8)	69.8 (1.6)	70.2 (1.3)	74.6 (1.4)	76.4 (1.2)	78.5 (1.2)	81.7 (1.3)	83.7 (1.2)
Vitamin D	40.3 (1.7)	39.8 (1.5)	48.6 (1.5)	49.8 (1.6)	61.0 (1.3)	63.4 (1.7)	66.9 (1.9)	74.8 (1.7)
Vitamin E	63.1 (1.4)	63.0 (1.6)	70.6 (1.0)	67.2 (1.1)	74.9 (1.2)	68.3 (0.9)	74.7 (1.3)	71.6 (1.1)
Vitamin K	61.2 (1.3)	74.7 (1.5)	72.0 (1.0)	78.1 (1.2)	75.3 (1.3)	79.4 (1.0)	76.2 (1.0)	82.3 (1.2)
**Total TNI score**	**63.4 (1.0)**	**66.7 (1.0)**	**70.0 (0.8)**	**70.9 (0.8)**	**75.8 (0.7)**	**75.0 (0.8)**	**75.7 (0.7)**	**78.0 (0.7)**
FNI component								
Calcium	81.3 (0.9)	73.1 (0.9)	83.5 (0.9)	75.2 (1.1)	81.4 (0.9)	64.0 (1.0)	69.9 (1.2)	59.8 (1.1)
Magnesium	71.1 (1.0)	74.3 (1.1)	74.6 (0.9)	77.1 (0.8)	75.0 (0.8)	76.7 (0.9)	69.2 (0.9)	72.1 (0.9)
Potassium	70.4 (1.0)	75.9 (1.0)	78.2 (0.9)	79.1 (0.8)	79.7 (0.7)	80.9 (0.8)	77.1 (1.0)	79.6 (1.1)
Choline	62.0 (1.1)	62.0 (1.0)	66.4 (0.8)	63.9 (0.9)	66.9 (0.9)	64.0 (0.8)	62.1 (1.1)	61.2 (0.9)
Vitamin A	56.1 (1.5)	63.1 (1.2)	60.4 (1.2)	66.7 (1.4)	62.1 (1.1)	67.6 (1.3)	68.9 (1.2)	73.4 (1.2)
Vitamin B12	–	–	–	–	93.6 (0.5)	87.9 (0.7)	93.9 (0.7)	87.0 (1.2)
Vitamin C	57.3 (1.7)	63.1 (1.5)	62.6 (1.2)	65.2 (1.4)	63.2 (1.1)	66.3 (1.3)	67.3 (1.4)	70.7 (1.5)
Vitamin D	30.0 (1.0)	23.6 (0.8)	30.1 (0.9)	25.3 (0.7)	31.5 (1.0)	25.8 (0.7)	27.1 (1.1)	20.7 (0.8)
Vitamin E	56.3 (1.1)	52.5 (1.4)	61.7 (1.0)	53.1 (0.9)	61.5 (0.9)	51.8 (1.2)	57.5 (1.0)	48.3 (1.0)
Vitamin K	60.3 (1.2)	73.6 (1.5)	70.0 (1.0)	75.9 (1.2)	71.7 (1.3)	76.1 (1.0)	71.3 (1.2)	77.4 (1.3)
**Total FNI score**	**60.5 (0.9)**	**62.3 (0.9)**	**65.2 (0.7)**	**64.6 (0.7)**	**68.6 (0.6)**	**66.1 (0.7)**	**66.4 (0.7)**	**65.0 (0.8)**

a The analytic sample includes individuals aged 19 years and older (N = 12 760) who were not pregnant and/or lactating and with complete and reliable information for at least 1 24-hr dietary recall and the Dietary Supplement and Prescription Medicine Questionnaire. NHANES Day 1 dietary weights were utilized to obtain estimates for the US population.

b Micronutrients included in this iteration of the TNI and FNI that were not components of the original, validated TNI and FNI are vitamins B12 (age-group dependent) and K.

c Although mean estimates reported in this table are nationally representative (ie, weighted estimates), the sample sizes reflect the analytic sample.

## Data Availability

Data described in the article are publicly available on the National Health and Nutrition Examination Survey website. The code book and analytic code will be made available upon request pending application and approval.

## RESEARCH SNAPSHOT

### Research Question:

How do micronutrient patterns, inclusive of intakes from foods, beverages, and dietary supplements, and their quality vary across the life course among US adults and children?

### Key Findings:

In this cross-sectional analysis of the US population (n = 19 903), adults and children exhibited widespread low micronutrient quality of the diet. Micronutrient quality, as assessed via Total Nutrient Index scores, varied by age group and was higher when inclusive of intakes from all sources (ie, foods/beverages and supplements), vs those from foods and beverages alone.
