# Supplementary material for: Application of the Total Nutrient Index as a Precision Nutrition Tool to Address Dietary Recommendations Across the Life Course
Source: J Acad Nutr Diet. Author manuscript; Available in PMC 2026 Sep 15. (PMC13575888; doi:10.1016/j.jand.2026.156368)
Supplement: Supplementary file 1 [file NIHMS2205793-supplement-Supplemental_File_Labeled_at_Figure_1.pdf]

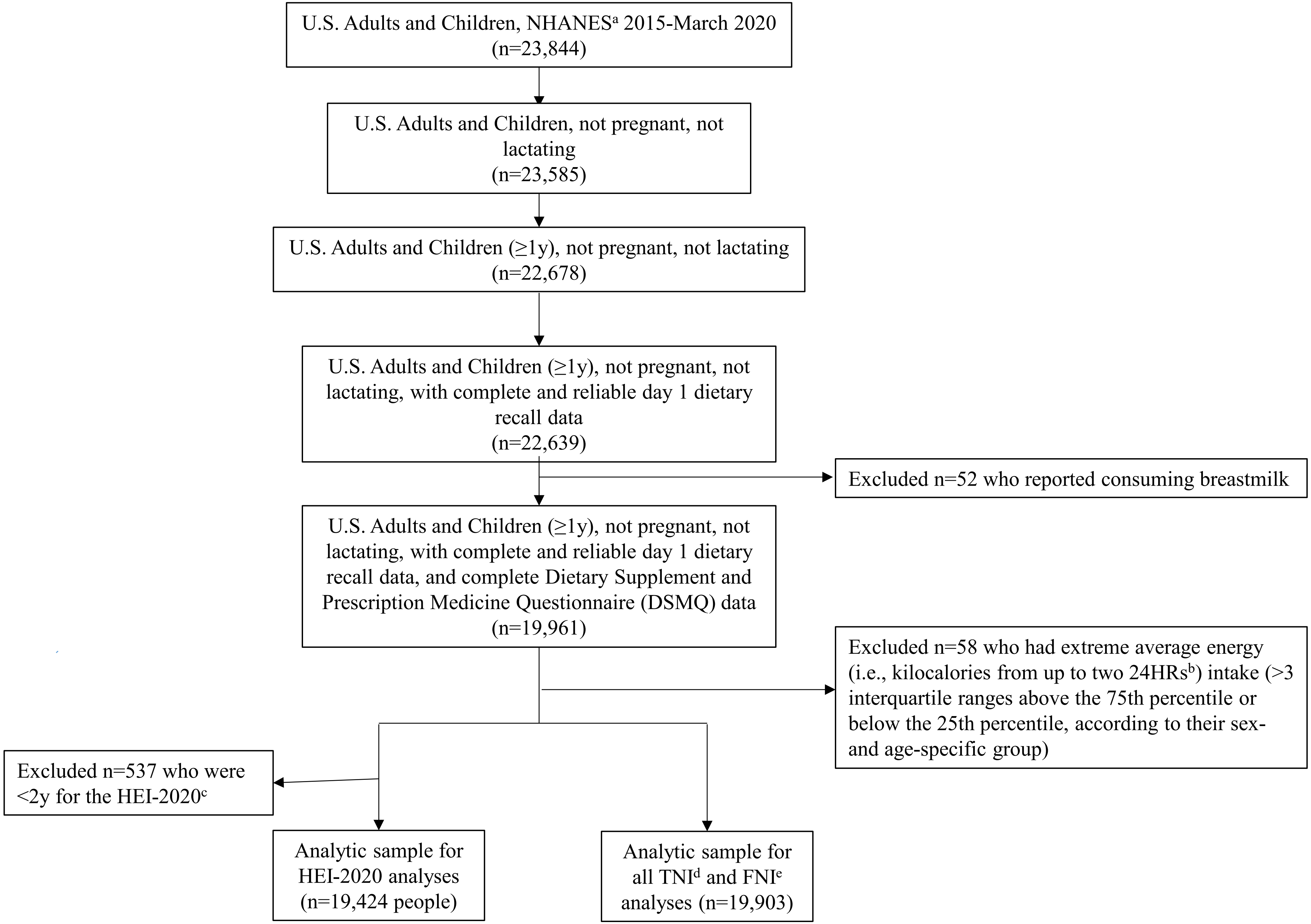
U.S. Adults and Children, NHANES[a] 2015-March 2020 (n=23,844)
U.S. Adults and Children, not pregnant, not lactating (n=23,585)
U.S. Adults and Children (≥1y), not pregnant, not lactating (n=22,678)
U.S. Adults and Children (≥1y), not pregnant, not lactating, with complete and reliable day 1 dietary recall data (n=22,639)
Excluded n=52 who reported consuming breastmilk
U.S. Adults and Children (≥1y), not pregnant, not lactating, with complete and reliable day 1 dietary recall data, and complete Dietary Supplement and Prescription Medicine Questionnaire (DSMQ) data (n=19,961)
Excluded n=58 who had extreme average energy (i.e., kilocalories from up to two 24HRs[b]) intake (>3 interquartile ranges above the 75th percentile or below the 25th percentile, according to their sex- and age-specific group)
Excluded n=537 who were <2y for the HEI-2020[c]
Analytic sample for HEI-2020 analyses (n=19,424 people)
Analytic sample for all TNI[d] and FNI[e] analyses (n=19,903)
